# Association of SARS-CoV-2 Infection With New-Onset Type 1 Diabetes Among Pediatric Patients From 2020 to 2021

**DOI:** 10.1001/jamanetworkopen.2022.33014

**Published:** 2022-09-23

**Authors:** Ellen K. Kendall, Veronica R. Olaker, David C. Kaelber, Rong Xu, Pamela B. Davis

**Affiliations:** 1 Center for Artificial Intelligence in Drug Discovery, Case Western Reserve University School of Medicine, Cleveland, Ohio; 2 The Center for Clinical Informatics Research and Education, The MetroHealth System, Cleveland, Ohio; 3 Center for Community Health Integration, Case Western Reserve University School of Medicine, Cleveland, Ohio

## Abstract

This cohort study assesses the association of COVID-19 with new-onset type 1 diabetes among pediatric patients.

## Introduction

Incidence of new-onset type 1 diabetes (T1D) increased during the COVID-19 pandemic,^1^ and this increase has been associated with SARS-CoV-2 infection.^2^ The US Centers for Disease Control and Prevention reported that pediatric patients with COVID-19 were more likely to be diagnosed with diabetes after infection, although types 1 and 2 were not separated.^3^ Therefore, whether COVID-19 was associated with new-onset T1D among youths remains unclear. This cohort study assessed whether there was an increase in new diagnoses of T1D among pediatric patients after COVID-19.

## Methods

Data were obtained using TriNetX Analytics Platform, a web-based database of deidentified electronic health records of more than 90 million patients, from the Global Collaborative Network, which includes 74 large health care organizations across 50 US states and 14 countries with diverse representation of geographic regions, self-reported race, age, income, and insurance types.^4^ The MetroHealth System institutional review board deemed the study exempt because it was determined to be non–human participant research. The study followed the STROBE reporting guideline.

The study population comprised pediatric patients in 2 cohorts: (1) patients aged 18 years or younger with SARS-CoV-2 infection between March 2020 and December 2021 and (2) patients aged 18 years or younger without SARS-CoV-2 infection but with non–SARS-CoV-2 respiratory infection during the same period. SARS-CoV-2 infection was defined as described in prior studies.^5^ These cohorts were subdivided into groups aged 0 to 9 years and 10 to 18 years.

Cohorts were propensity score matched (1:1 using nearest-neighbor greedy matching) for demographics and family history of diabetes (Table). Risk of new diagnosis of T1D within 1, 3, and 6 months after infection were compared between matched cohorts using hazard ratios (HRs) and 95% CIs. Statistical analyses were conducted in the TriNetX Analytics Platform. Further details and analyses from the TriNetX database are given in the eMethods in the Supplement.

**Table.** Characteristics of Patient Cohorts Before and After Propensity Score Matching

Characteristic	Before matching^a^		SMD^b^	After matching^a^		SMD^b^
	COVID-19 (n = 314 917)	Other respiratory infections (n = 776 577)		COVID-19 (n = 285 628)	Other respiratory infections (n = 285 628)	
Age when data accessed, mean (SD), y	11.1 (5.8)	7.2 (5.3)	0.70	10.3 (5.6)	10.3 (5.5)	0.01
Age at index, mean (SD), y^c^	10.0 (5.8)	6.2 (5.3)	0.69	9.3 (5.5)	9.2 (5.5)	0.01
Sex						
Female	157 078 (49.9)	379 017 (48.8)	0.02	142 288 (49.8)	141 538 (49.6)	0.01
Male	157 731 (50.1)	397 445 (51.2)	0.02	143 289 (50.2)	144 029 (50.4)	0.01
Ethnicity^d^						
Hispanic/Latinx	52 244 (16.6)	123 597 (15.9)	0.02	46 055 (16.1)	47 897 (16.8)	0.02
Not Hispanic/Latinx	175 828 (55.8)	427 820 (55.1)	0.02	160 514 (56.2)	160 605 (56.2)	0.001
Unknown	86 845 (27.6)	225 160 (29)	0.03	79 059 (27.7)	77 126 (27)	0.02
Race^d^						
American Indian or Alaska Native	1013 (0.3)	3099 (0.4)	0.01	995 (0.3)	930 (0.3)	0.004
Asian	5692 (1.8)	18 790 (2.4)	0.04	5564 (1.9)	5197 (1.8)	0.01
Black or African American	53 491 (17)	120 709 (15.5)	0.04	45 124 (15.8)	48 355 (16.9)	0.03
Native Hawaiian or Other Pacific Islander	433 (0.1)	998 (0.1)	0.002	387 (0.1)	338 (0.1)	0.01
White	167 723 (53.3)	402 264 (51.8)	0.03	154 078 (53.9)	153 194 (53.6)	0.01
Unknown	86 565 (27.5)	230 717 (29.7)	0.05	79 480 (27.8)	77 614 (27.2)	0.02
Family history of diabetes	6961 (2.2)	6815 (0.9)	0.11	4313 (1.5)	5342 (1.9)	0.03

Abbreviation: SMD, standardized mean difference.

^a^ Data are presented as number (percentage) of patients unless otherwise indicated.

^b^ An SMD greater than 0.1 is a threshold recommended for declaring imbalance.

^c^ Index event was diagnosis of COVID-19 or other respiratory infection.

^d^ Race and ethnicity were self-reported and included because they are reported to be associated with COVID-19 diagnosis and severity.

## Results

The Table shows population characteristics before and after matching. The study population included 1 091 494 pediatric patients: 314 917 with COVID-19 and 776 577 with non–COVID-19 respiratory infections. The matched cohort included 571 256 pediatric patients: 285 628 with COVID-19 and 285 628 with non–COVID-19 respiratory infections. By 6 months after COVID-19, 123 patients (0.043%) had received a new diagnosis of T1D, but only 72 (0.025%) were diagnosed with T1D within 6 months after non–COVID-19 respiratory infection. At 1, 3, and 6 months after infection, risk of diagnosis of T1D was greater among those infected with SARS-CoV-2 compared with those with non–COVID-19 respiratory infection (1 month: HR, 1.96 [95%CI, 1.26-3.06]; 3 months: HR, 2.10 [95% CI, 1.48-3.00]; 6 months: HR, 1.83 [95% CI, 1.36-2.44]) and in subgroups of patients aged 0 to 9 years, a group unlikely to develop type 2 diabetes, and 10 to 18 years (Figure). Similar increased risks were observed among children infected with SARS-CoV-2 compared with other control cohorts at 6 months (fractures: HR, 2.09 [95% CI, 1.41- 3.10]; well child visits: HR, 2.10 [95% CI, 1.61- 2.73]).

**Figure.**
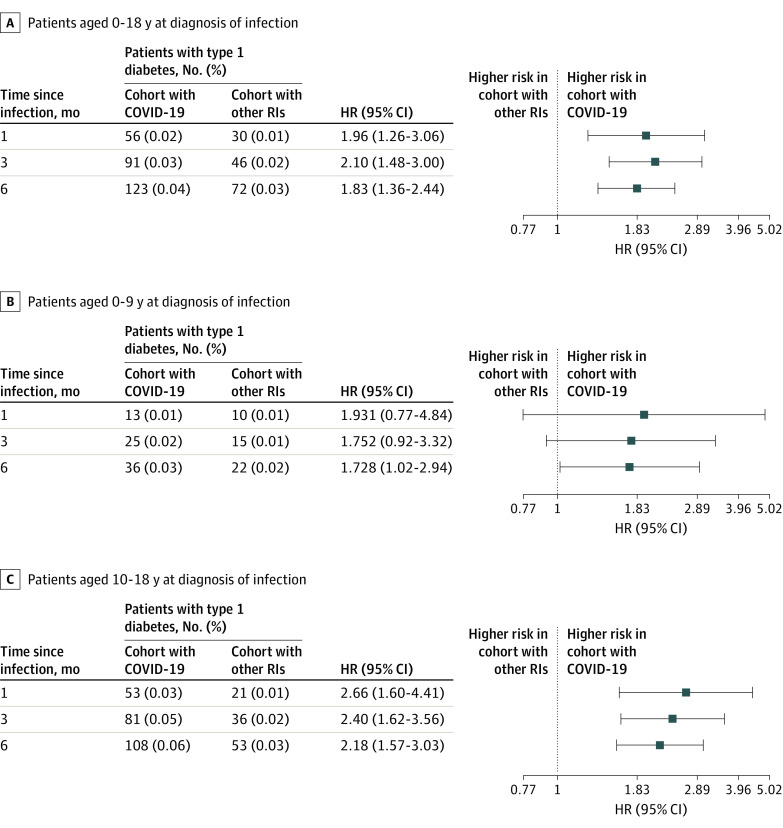
Comparison of Risk of New Diagnosis of Type 1 Diabetes in Patients After COVID-19 vs Other Respiratory Infections (RIs) HR indicates hazard ratio.

## Discussion

In this study, new T1D diagnoses were more likely to occur among pediatric patients with prior COVID-19 than among those with other respiratory infections (or with other encounters with health systems). Respiratory infections have previously been associated with onset of T1D,^6^ but this risk was even higher among those with COVID-19 in our study, raising concern for long-term, post–COVID-19 autoimmune complications among youths. Study limitations include potential biases owing to the observational and retrospective design of the electronic health record analysis, including the possibility of misclassification of diabetes as type 1 vs type 2, and the possibility that additional unidentified factors accounted for the association. Results should be confirmed in other populations. The increased risk of new-onset T1D after COVID-19 adds an important consideration for risk-benefit discussions for prevention and treatment of SARS-CoV-2 infection in pediatric populations.

## Notes

Supplement.
eMethods.

## References

[1] Gottesman BL, Yu J, Tanaka C, Longhurst CA, Kim JJ. Incidence of new-onset type 1 diabetes among US children during the COVID-19 global pandemic. JAMA Pediatr. 2022;176(4):414-415. doi:10.1001/jamapediatrics.2021.5801 35072727 PMC8787677

[2] Qeadan F, Tingey B, Egbert J, . The associations between COVID-19 diagnosis, type 1 diabetes, and the risk of diabetic ketoacidosis: a nationwide cohort from the US using the Cerner real-world data. PLoS One. 2022;17(4):e0266809. doi:10.1371/journal.pone.0266809 35439266 PMC9017888

[3] Barrett CE, Koyama AK, Alvarez P, . Risk for newly diagnosed diabetes >30 days after SARS-CoV-2 infection among persons aged <18 years—United States. MMWR Morb Mortal Wkly Rep. 2022;71(2):59-65. doi:10.15585/mmwr.mm7102e2 35025851 PMC8757617

[4] TriNetX. Home page. Accessed April 4, 2022. https://trinetx.com/

[5] Taquet M, Geddes JR, Husain M, Luciano S, Harrison PJ. 6-month neurological and psychiatric outcomes in 236 379 survivors of COVID-19: a retrospective cohort study using electronic health records. Lancet Psychiatry. 2021;8(5):416-427. doi:10.1016/S2215-0366(21)00084-5 33836148 PMC8023694

[6] Lönnrot M, Lynch KF, Elding Larsson H, ; TEDDY Study Group. Respiratory infections are temporally associated with initiation of type 1 diabetes autoimmunity: the TEDDY study. Diabetologia. 2017;60(10):1931-1940. doi:10.1007/s00125-017-4365-5 28770319 PMC5697762

