# Supplementary material for: Association of SARS-CoV-2 Infection With New-Onset Type 1 Diabetes Among Pediatric Patients From 2020 to 2021
Source: JAMA Netw Open. 2022 Sep 23;5(9):e2233014. doi: 10.1001/jamanetworkopen.2022.33014 (PMC9508649; doi:10.1001/jamanetworkopen.2022.33014)
Supplement: Supplement. — eMethods. [file jamanetwopen-e2233014-s001.pdf]

# Supplemental Online Content

Kendall EK, Olaker VR, Kaelber DC, Xu R, Davis PB. Association of SARS-CoV-2 infection with new-onset type 1 diabetes among pediatric patients from 2020 to 2021. *JAMA Netw Open*. 2022;5(9):e2233014. doi:10.1001/jamanetworkopen.2022.33014

**eMethods.**

This supplemental material has been provided by the authors to give readers additional information about their work.

© 2022 Kendall EK et al. *JAMA Network Open.*

**eMethods.**

**TriNetX Database and Statistical Analysis Description of TriNetX database**: The data used in this study was accessed on April 4, 2022 from the TriNetX COVID-19 Global Collaborative Network. This resource provides access to electronic health records (EHRs) (diagnoses, procedures, medications, laboratory values, genomic information) from over 90 million patients from 74 healthcare organizations, which is de-identified per criteria from the Health Insurance Portability and Accountability Act (HIPAA), Section §164.514(a) of the HIPAA Privacy Rule. MetroHealth System, Cleveland, Ohio, IRB has determined any research using TriNetX, is not Human Subject Research and therefore exempt from IRB review.

The TriNetX platform de-identifies and aggregates EHR data from 74 contributing healthcare systems, most of which are large academic medical institutions with both inpatient and outpatient facilities at multiple locations, across 50 states in the US and 14 countries. Patient EHR data includes information from hospitals, primary care, and specialty treatment providers, covering diverse geographic locations, age groups, racial and ethnic groups, income levels and insurance types including various commercial insurances, governmental insurance (Medicare and Medicaid), self-pay/uninsured, worker compensation insurance, military/VA insurance among others. Race and ethnicity data in TriNetX is derived from self-reports in the clinical EHR systems, which is then mapped to the following categories: (1) Race: Asian, American Indian or Alaskan Native, Black or African American, Native Hawaiian or Other, White, Unknown race; and (2) Ethnicity: Hispanic or Latino, Not Hispanic or Latino, Unknown Ethnicity.

**Statistical analysis:** The status of SARS-CoV-2 infection was based on the lab-test confirmed presence or the International Classification of Diseases (ICD-10) diagnosis codes (complete cohort terms defined below). The outcome measure of type 1 diabetes (T1D) was determined by the presence of ICD-10 code E10 (“Type 1 diabetes mellitus”). SARS-CoV-2 infections that were diagnosed between March 11, 2020 and December 1, 2021 were recorded for children under age 18 years. We also established a cohort of

© 2022 Kendall EK et al. *JAMA Network Open.*

children diagnosed with other respiratory infection (cohort terms below) who were never diagnosed with SARS-CoV2 during the same time period. Each cohort was matched with the cohort of children with SARS-CoV2 infection by the TriNetX built-in propensity score matching function (1:1 matching using a nearest neighbor greedy matching algorithm with a caliper of 0.25 times the standard deviation). Risk of new diagnosis of type 1 diabetes following date of infection was then compared for the SARS-CoV2 cohort to the other respiratory infection cohort (or the fracture or routine visit cohorts) using hazard ratios and 95% confidence intervals. Kaplan-Meier analysis was used to estimate the probability of clinical outcomes. Cox's proportional hazards model was used to compare the two matched cohorts. The proportional hazard assumption was tested using the generalized Schoenfeld approach. For sensitivity analysis, we also created two additional control cohort of children: (1) who had broken bones during this period but were never diagnosed with SARS-CoV2 infection, and (2) who had routine encounters with the health care system but were never diagnosed with SARS-CoV2. Risks for new diagnosis of T1D were compared between children with SARS-CoV-2 infection and these control cohorts as described above. The TriNetX Platform calculates the hazard ratios and associated confidence intervals, using R's Survival package v3.2-3. For generating hazard ratios, TriNetX sets robust=FALSE using the R survival package, but it does not take into account potential clustering of COVID-19 cases within the healthcare organizations or specific geolocations, a potential weakness or confounding factor in the analysis.

The inclusion criteria for the COVID-19 cohorts are found below and include a mix of diagnostic codes and positive laboratory tests. This same list was also used as exclusion criteria for the control cohorts. Cohort terms were adopted from ref 5.

U07.1: COVID-19

U07.2: COVID-19, virus not identified (WHO)

J12.81: Pneumonia due to SARS-associated coronavirus

© 2022 Kendall EK et al. *JAMA Network Open.*

B97.29: Other coronavirus as the cause of diseases classified elsewhere

B34.2: Coronavirus infection, unspecified

9088: Positive SARS coronavirus 2 and related RNA

94500-6: SARS-CoV-2 (COVID-19) RNA [presence] in Respiratory specimen by NAA with probe detection

94309-2: SARS-CoV-2 (COVID-19) RNA [Presence] in Specimen by NAA with probe detection

94534-5: SARS-CoV-2 (COVID-19) RdRp gene [Presence] in Respiratory specimen by NAA with probe detection

94565-9: SARS-CoV-2 (COVID-19) RNA [Presence] in Nasopharynx by NAA with non-probe detection

94315-9: SARS-related coronavirus E gene [Presence] in Specimen by NAA with probe detection

94759-8: SARS-CoV-2 (COVID-19) RNA [Presence] in Nasopharynx by NAA with probe detection

94316-7: SARS-CoV-2 (COVID-19) N gene [Presence] in Specimen by NAA with probe detection

94559-2: SARS-CoV-2 (COVID-19) ORF1ab region [presence] in Respiratory specimen by NAA with probe detection

95209-3: SARS-CoV+SARS-CoV-2 (COVID-19) Ag [Presence] in Respiratory specimen by Rapid immunoassay

94558-4: SARS-CoV-2 (COVID-19) Ag [Presence] in Respiratory specimen by Rapid immunoassay

95608-6: SARS-CoV-2 (COVID-19) RNA [Presence] in Respiratory specimen by NAA with non-probe detection

96119-3: SARS-CoV-2 (COVID—19) Ag [Presence] in Upper respiratory specimen by immunoassay

94763-0: SARS-CoV-2 (COVID-19) [Presence] in Specimen by Organism specific culture

© 2022 Kendall EK et al. *JAMA Network Open.*

94533-7: SARS-CoV-2 (COVID-19) N gene [Presence] in Respiratory specimen by NAA with probe detection

94760-6: SARS-CoV-2 (COVID-19) N gene [presence] in Nasopharynx by NAA with probe detection

95406-5: SARS-CoV-2 (COVID-19) RNA [presence] in Nose by NAA with probe detection

94845-5: SARS-CoV-2 (COVID-19) RNA [presence] in Saliva (oral fluid) by NAA with probe detection

94758-0: SARS-related coronavirus E gene [Presence] in Respiratory specimen by NAA with probe detection

96763-8: SARS-CoV-2 (COVID-19) E gene [presence] in Respiratory specimen by NAA with probe detection

94502-2: SARS-related coronavirus RNA [Presence] in Respiratory specimen by NAA with probe detection

94314-2: SARS-CoV-2 (COVID-19) RdRp gene [presence] in Specimen by NAA with probe detection

96123-5: SARS-CoV-2 (COVID-19) RdRp gene [presence] in Upper respiratory specimen by NAA with probe detection

97097-0: SARS-CoV-2 (COVID-19) Ag [presence] in Upper respiratory specimen by Rapid immunoassay

94647-5: SARS-related coronavirus RNA [Presence] in Specimen by NAA with probe detection

95409-9: SARS-CoV-2 (COVID-19) N gene [presence] in Nose by NAA with probe detection

The other respiratory infection control cohort was defined as having any of the following diagnostic codes within the index event time window:

J00: Acute nasopharyngitis [common cold]

J01: Acute sinusitis

© 2022 Kendall EK et al. *JAMA Network Open.*

J02: Acute pharyngitis

J03: Acute tonsilitis

J04: Acute laryngitis and tracheitis

J05: Acute obstructive laryngitis [croup] and epiglottitis

J06: Acute upper respiratory infections of multiple and unspecified sites

J09-J18: Influenza and Pneumonia

J20-J22: Other acute lower respiratory infections

The well child visit cohort was defined by:

| | |
|---|---|
| ICD10CM:Z00.12 | Encounter for routine child health examination (between 0 and 18 years old at event) |

The fracture cohort was defined by:

| | |
|---|---|
| ICD10CM:S32 | Fracture of lumbar spine and pelvis (between 0 and 18 years old at event) |
| ICD10CM:S72 | Fracture of femur (between 0 and 18 years old at event) |
| ICD10CM:S82 | Fracture of lower leg, including ankle (between 0 and 18 years old at event) |
| ICD10CM:S52 | Fracture of forearm (between 0 and 18 years old at event) |
| ICD10CM:S42 | Fracture of shoulder and upper arm (between 0 and 18 years old at event) |

© 2022 Kendall EK et al. *JAMA Network Open.*
